# Non-synaptic Plasticity in Leech Touch Cells

**DOI:** 10.3389/fphys.2019.01444

**Published:** 2019-11-27

**Authors:** Sonja Meiser, Go Ashida, Jutta Kretzberg

**Affiliations:** ^1^Computational Neuroscience, Department of Neuroscience, Faculty VI, Carl von Ossietzky University of Oldenburg, Oldenburg, Germany; ^2^Cluster of Excellence Hearing4all, Department of Neuroscience, Faculty VI, Carl von Ossietzky University of Oldenburg, Oldenburg, Germany

**Keywords:** invertebrate, mechanoreceptor, sodium–potassium pump, Hodgkin–Huxley neuron model, M-type K^+^ current, spike count, resting potential, input resistance

## Abstract

The role of Na^+^/K^+^-pumps in activity-dependent synaptic plasticity has been described in both vertebrates and invertebrates. Here, we provide evidence that the Na^+^/K^+^-pump is also involved in activity-dependent non-synaptic cellular plasticity in leech sensory neurons. We show that the resting membrane potential (RMP) of T cells hyperpolarizes in response to repeated somatic current injection, while at the same time their spike count (SC) and the input resistance (IR) increase. Our Hodgkin–Huxley-type neuron model, adjusted to physiological T cell properties, suggests that repetitive action potential discharges lead to increased Na^+^/K^+^-pump activity, which then hyperpolarizes the RMP. In consequence, a slow, non-inactivating current decreases, which is presumably mediated by voltage-dependent, low-threshold potassium channels. Closing of these putative M-type channels due to hyperpolarization of the resting potential increases the IR of the cell, leading to a larger number of spikes. By this mechanism, the response behavior switches from rapidly to slowly adapting spiking. These changes in spiking behavior also effect other T cells on the same side of the ganglion, which are connected via a combination of electrical and chemical synapses. An increased SC in the presynaptic T cell results in larger postsynaptic responses (PRs) in the other T cells. However, when the number of elicited presynaptic spikes is kept constant, the PR does not change. These results suggest that T cells change their responses in an activity-dependent manner through non-synaptic rather than synaptic plasticity. These changes might act as a gain-control mechanism. Depending on the previous activity, this gain could scale the relative impacts of synaptic inputs from other mechanoreceptors, versus the spike responses to tactile skin stimulation. This multi-tasking ability, and its flexible adaptation to previous activity, might make the T cell a key player in a preparatory network, enabling the leech to perform fast behavioral reactions to skin stimulation.

## Introduction

Understanding the mechanisms of how sensory information elicits behavioral reactions is a major goal in neuroscience. The experimentally easily amenable nervous system of the medicinal leech makes this animal a useful model organism to investigate the neuronal basis of sensory processing (Kristan et al., 2005; Wagenaar, 2015). Touching a leech’s skin triggers a behavioral response with a surprisingly high accuracy – the local bend (Kristan et al., 1982). The animal can distinguish between two touch locations just as precisely as the human fingertip (Johnson, 2001; Baca et al., 2005; Thomson and Kristan, 2006; Pirschel and Kretzberg, 2016). The leech central nervous system (CNS) contains a highly repetitive ventral nerve cord with one ganglion per segment. Each ganglion contains an ensemble of around 400 mostly paired neurons serving as the basis of diverse sensory-input motor-output networks (Kristan et al., 2005). The sensory input layer consists of three different types of leech mechanosensory cells [touch (T), pressure (P), and nociceptive (N) cells], which share several fundamental properties with the human tactile receptors (Baca et al., 2005; Smith and Lewin, 2009; Pirschel and Kretzberg, 2016). Early studies focused mostly on P cells, because stimulation of a single P cell is sufficient to elicit muscle movements for several behavioral responses, like swimming or local bending (Kristan, 1982; Kristan et al., 1982). Since T and N cells showed only minor contributions to these movements, they were not further investigated. However, Pirschel and Kretzberg (2016) suggested that T cells might play a substantial role in the local bend response, making in-depth investigations on these sensory neurons necessary.

The current study focuses on these T cells, which are low threshold, rapidly adapting sensory neurons. They primarily encode the temporal qualities, especially velocity, of applied mechanosensory stimuli during the onset and offset phases of the stimulation (Nicholls and Baylor, 1968b; Carlton and McVean, 1995). There are three bilateral pairs of T cells in one ganglion which form both electrical and chemical synaptic connections with each other (Nicholls and Baylor, 1968b; Baylor and Nicholls, 1969b; Li and Burrell, 2008). Moreover T cells receive polysynaptic input from the other mechanoreceptor type (P cells) and nociceptors (N cells), leading to a combination of excitatory and inhibitory potentials (Burgin and Szczupak, 2003). Several long-range dendritic processes of T cells run through the ipsilateral nerve roots in the body wall to branch extensively in the base of the layer of epithelial cells and end at a few micrometers from the skin surface (Blackshaw, 1981). The rapidly adapting human Meissner corpuscles show similar response properties, elicited by encapsulated unmyelinated nerve endings with stretch-sensitive ion channels in the tip. Potentially, the nerve endings of T cells may also contain these mechanosensitive channels, which may change opening probability after repeated stimulation, like in human hair cells during stimulation with a high sound pressure level (Hakizimana et al., 2012). Because the entire extent of arborization of one T cell spans three segmental ganglia, each cell responds to touch of the skin at its own and the adjacent anterior and posterior segments (Yau, 1976). The receptive fields of each T cell cover either the dorsal, lateral, or ventral skin area on one side (Nicholls and Baylor, 1968b). Like other invertebrate neurons, T cells are unipolar, meaning that dendrites and axon are not clearly separated, but form a continuum of processes (Rolls and Jegla, 2015). Moreover, like several invertebrate neurons (Calabrese, 1980; Meyrand et al., 1992), leech T cells were found to have at least two distinct spike-initiation zones. A peripheral spike initiation zone near the skin conveys information about touch stimuli, and a central one close to the soma processes synaptic inputs within the ganglion (Burgin and Szczupak, 2003; Kretzberg et al., 2007).

High frequency spiking in touch mechanoreceptors triggered by somatic electrical stimulation or peripheral skin stimulation (Baylor and Nicholls, 1969a) induces a long-term afterhyperpolarization (AHP), arising from the activation of the Na^+^/K^+^-pump (also called the Na^+^/K^+^-ATPase) and a Ca^2+^-dependent K^+^ current (Nicholls and Baylor, 1968a; Baylor and Nicholls, 1969a; Jansen and Nicholls, 1973; Scuri et al., 2002, 2007). Previous studies pointed out that modulation of the Na^+^/K^+^-pump activity is involved in activity-dependent synaptic plasticity between two ipsilateral T neurons (Catarsi and Brunelli, 1991; Catarsi et al., 1993; Scuri et al., 2002, 2007; Lombardo et al., 2004). Additionally, high-frequency stimulation of a T cell elicits long-term depression in the activated pathway and potentiation in the non-activated T cell synapses (Burrell and Sahley, 2004). Furthermore, low-frequency stimulation of T cells can depress synapses through an endocannabinoid-dependent mechanism, which has also been observed in the mammalian spinal cord (Li and Burrell, 2009, 2010).

Here, we show that T cell activity is also influenced by non-synaptic plasticity. Based on our electrophysiological experiments and modeling approaches, we demonstrate that repeated somatic T cell stimulation enhances Na^+^/K^+^-pump activity, which gradually hyperpolarizes the resting membrane potential (RMP). In consequence, a slow, non-inactivating (putative M-type K^+^) current decreases, resulting in a higher input resistance (IR) and a larger number of tonic spikes. Furthermore, our T cell double recordings show that the Na^+^/K^+^-pump is also involved in activity-dependent non-synaptic cellular plasticity among leech sensory neurons. Our recording results indicate that the increase in presynaptic spike count (SC) due to non-synaptic plasticity also affects the PR in another ipsilateral T cell. However, this effect is not specific to stimulus history, indicating non-synaptic rather than synaptic plasticity.

## Materials and Methods

### Animals and Preparation

**FIGURE 1:**
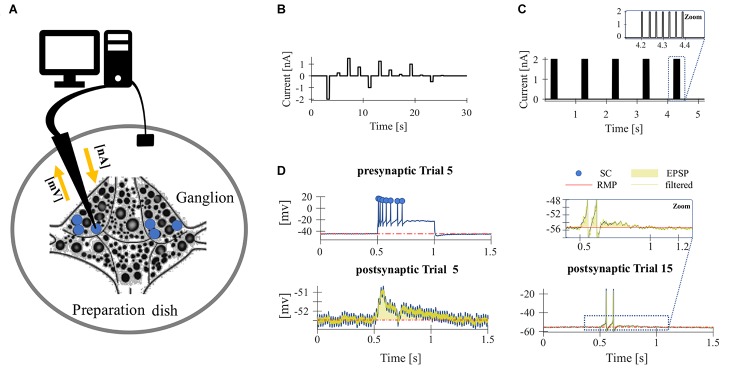
Experimental design. **(A)** Sketch of the isolated ganglia preparation and the principle of the current-clamp recording. The membrane potential of one of the T cells (blue) was recorded with an intracellular electrode (black arrow). **(B,C)** Stimulus protocol for repeated electrical soma stimulation. Each experiment consisted of 15–20 identical trial repetitions. **(B)** For the experiments presented in Figures 2, 4A,B, 12 electrical pulses of different current amplitudes were injected into the T cell soma. **(C)** For studying synaptic interaction of T cells (Figures 4C–F), five pulse packages were injected into the soma if one T cell. Each package contained a fixed number (1–7) of pulses that elicits the same number of single action potentials. The zoom inset shows a package with 7 pulses. **(D)** The neuronal responses were quantified by the following features: (presynaptic) spike count (SC, blue dots indicate counted spikes): total number of spikes elicited by the neuron and recorded in the soma between the stimulus onset (0.5 s) and offset (1 s); resting membrane potential (RMP, red): averaged membrane potential in the 2.5 s prior to the first current pulse; postsynaptic response (PR): averaged difference between the filtered recorded membrane potential and the RMP calculated from the start to 200 ms after the end of the presynaptic current stimulus (yellow transparent area). Synaptic potentials sometimes triggered spikes in the postsynaptic cell (see Trial 15 for an example), but not in all (see Trial 5 for an example). The calculation of PR included spikes if they were elicited.

The experiments were performed on adult hermaphrodite medicinal leeches (*Hirudo verbana*) obtained from the Biebertaler Leech Breeding Farm (Biebertal, HE, Germany). According to German regulations, no approval of an ethics committee was required for the work on these invertebrates. The animals were kept at room temperature in tanks with ocean sea-salt diluted with purified water (1:1000). All experiments were performed at room temperature. The leeches were anesthetized with ice-cold saline (mM: 115 NaCl, 4 KCl, 1.8 CaCl_2_, 10 Glucose, 4.6 Tris–maleate, 5.4 Tris base and buffered at pH 7.4 with NaOH, modified after Muller and Scott, 1981) before and during dissection. We used isolated ganglia, dissected from segments 9–13 and pinned them, ventral side up, to a plastic petri dish, coated with the silicone elastomer *Sylgard* (Dow Corning Corporation, Midland, MI, United States) (Figure 1A).

### Electrophysiological Technique

The experimental rig consisted of two mechanical micromanipulators type MX-1 (TR 1, Narishige, Tokyo, Japan) and two amplifiers (SEC-05X, NPI Electronic, Tamm, Germany) (Kretzberg et al., 2016). Neuronal responses were recorded (sample rate 100 kHz) and analyzed using custom-written MATLAB software (MATLAB 9.1-9.5, MathWorks, Natick, MA, United States). We performed intracellular single and double recordings from mechanosensory touch cells, while injecting current into one T cell soma. For these current clamp recordings, the cell soma was impaled with borosilicate microelectrodes (TW100F-4, World Precision Instruments Inc., Sarasota, FL, United States) pulled with the micropipette puller P97 Flaming Brown (Sutter Instruments Company, Novato, CA, United States). The glass electrodes were filled with 3 M potassium acetate and had resistances of 15–30 MΩ. The neurons were identified by the size and the location of their cell bodies with a binocular microscope (Olympus szx7, Olympus, Tokyo, Japan) as well as by their firing pattern (Nicholls and Baylor, 1968b).

### Experimental Design

To investigate the effect of repeated mechanoreceptor stimulation on the physiological properties of T cells and their synaptic partners we used somatic current injection. Intracellular single recordings of T cells in isolated ganglia were performed by stimulating the neuron in each trial with a series of 12 current pulses in a pseudo-randomized order (Figure 1B). The amplitude of the pulses varied between −2 and +1.5 nA. The duration of each pulse was 500 ms and the inter-pulse-interval was 2.5 s long. The inter-trial-interval was 5 s long. Each experiment consisted of 15–20 identical trial repetitions. While injecting current into the T cell soma with the intracellular electrode, we recorded the membrane potential of the stimulated T cell with the same electrode.

To analyze the synaptic connections between T cells, we performed ipsilateral double recordings and recorded from two of the three T cells in all combinations. While injecting current into one T cell soma (presynaptic) with one electrode, we recorded the membrane potential of the unstimulated T cell (postsynaptic) with a second electrode. In addition to the protocol shown in Figure 1B, a pulse-package protocol (Figure 1C) was applied. One pulse-package comprised a fixed number (1–7) of short current pulses (2 nA, 5 ms), which were injected into the T cell soma to trigger one action potential each. In consequence, the pulse-package protocol elicited the same number and timing of presynaptic action potentials in each trial. Each trial consisted of five pulse packages and each sub-experiment was composed of 25 trial repetitions. The pause between the single pulses in a package was 30 ms and the starting times of the packages were always separated by 1 s (independent of the number of pulses in the package).

In total, we analyzed 20 single and 23 double recordings (9 with 500 ms current pulse injections, 14 with single current pulse injections) of T cells. During the recordings, the electrode properties usually changed slightly, leading to an average increase in electrode resistance by +7 MΩ, and an average electrode offset drift by −4 mV. Five additional single recordings were excluded from the analysis, because the electrode resistance changed by more then10 MΩ due to clogging, or because the electrode offset drifted by more than −6.25 mV.

### Data Analysis

The neuronal responses of the stimulated T cells and their synaptic partner neurons were quantified by the following response features (Figure 1D): SC, RMP, cell IR, and postsynaptic response (PR).

• **SC** [spike number] was defined as the total number of spikes elicited by the neuron and recorded in the soma during the 500 ms between the stimulus onset and offset. Spike detection was accomplished using custom-developed MATLAB software. Spikes were defined by the following parameters: minimum threshold [mV], minimum duration [ms], and minimum spike amplitude [mV]. A spike was detected when the membrane potential depolarized by the minimum spike threshold and the relative spike height, from rest to peak, was at least as high as the minimum spike amplitude. Additionally, a peak was only accepted as an action potential if the detected peak at half of the prominence had the required minimum duration.
• **RMP** [mV] of each trial (*U*_rest_) was computed as the averaged membrane potential in the 2.5 s before the first current pulse starts.
• **IR** [MΩ] was calculated based on the average membrane potential (*U*_stim_) in response to a 500 ms long hyperpolarizing current pulse of *I* = −1 nA to avoid the influence of active processes. It was calculated with Ohm’s law: *R* [MΩ] = (*U*_stim_ [mV] − *U*_rest_ [mV])/*I* [nA].
• **PR** [mV] was calculated as average difference between the recorded membrane potential and the RMP of the postsynaptic (unstimulated) cell. The potential difference was averaged in the period from the start to 200 ms after the end of the presynaptic current stimulus, no matter if spikes were elicited postsynaptically or not. To reduce the noise caused by electric hum superimposed to the recorded postsynaptic signal, we used a notch filter which removed at least half the power of the frequency components in the range of 47–53 Hz.

**FIGURE 2:**
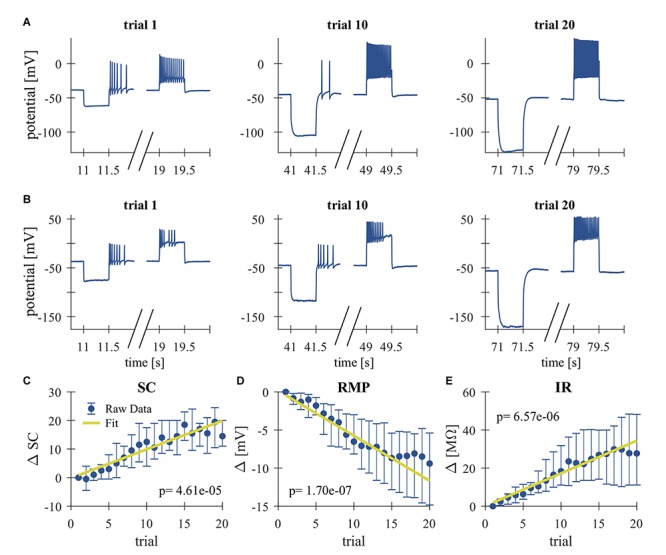
Repeated somatic current injection effects physiological properties of T cells. **(A,B)** Representative responses of intracellularly recorded T cells to a 500 ms long somatic current pulse injection of –1 nA (seconds 11–11.5, 41–41.5, and 71–71.5), which was used for the calculation of the IR and often elicited rebound spikes, and +1 nA (seconds 19–19.5, 49–49.5, and 79–79.5) in Trial 1, 10, 20. **(A)** Example of a slowly adapting initial response, **(B)** Example of a rapidly adapting initial response. **(C–E)** Dependencies of changes in response parameters on trial repetitions shown as median and quartiles of all T cells (*N* = 20). Linear fits were calculated for each cell individually and the obtained slopes were tested to differ significantly from 0 (*t*-test, α = 0.05, *p*-values given in the panels). The yellow line indicates the average of the fits for all cells. **(C)** Spike count (SC) difference from first trial (absolute number). **(D)** Resting membrane potential (RMP) difference from first trial (mV). **(E)** Input resistance difference from first trial (MΩ).

In the results section, response changes (ΔSC, ΔRMP, ΔIR, ΔPR) caused by repeated stimulation are displayed as differences in the measured response features (SC, RMP, IR, PR) between the N_th_ and the first trial repetition. The observed distributions of these response feature changes are reported as median, and first (Q1) and third (Q3) quartiles in the figures. To show the increase (ΔSC, ΔIR, ΔPR) or decrease (ΔRMP) over trial repetitions, linear fits were calculated for each cell individually. The obtained slopes were tested to differ significantly from 0 (*t*-test, α = 0.05). In Figures 2, 4, the average of the linear fits for all cells indicates if the response features increased or decreased with trial repetitions.

**FIGURE 3:**
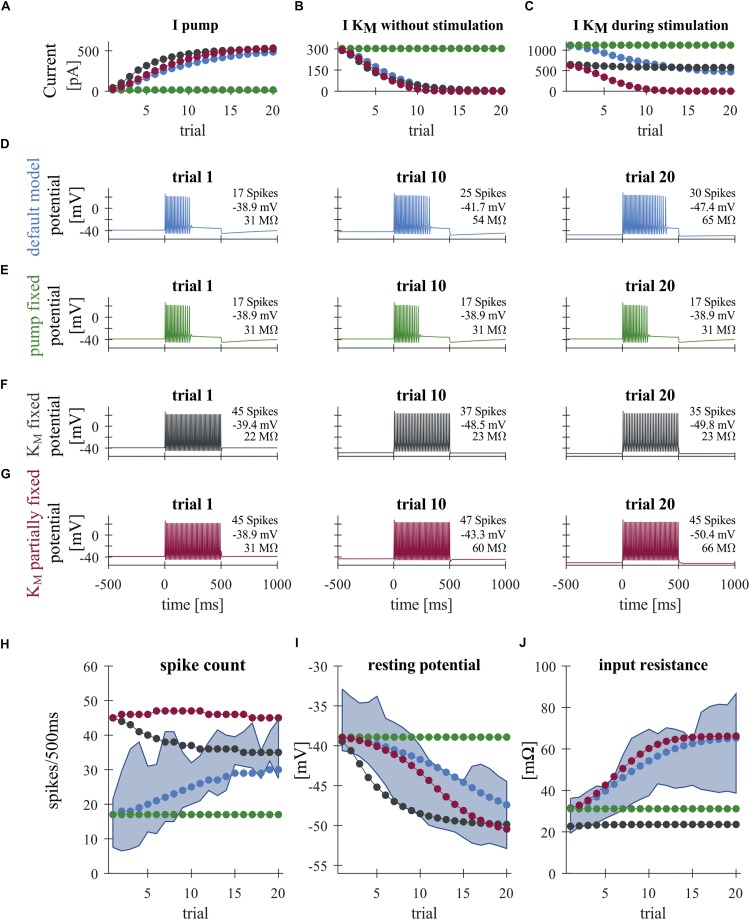
Modeling T cell spike responses. **(A)** Average Na^+^/K^+^-pump current during +1 nA stimulation. **(B)** Average M-type K^+^ current at 0–500 ms before the +1 nA stimulation. **(C)** Average M-type K^+^ current during +1 nA stimulation. The K_M_ current is generally larger during stimulation **(C)** than without stimulation **(B)**, because of the increased average driving voltage (*E_*K*_–V*) associated with spiking. **(D–G)** Responses of different T cell models to a current injection of +1 nA in Trials 1, 10 and 20. See Figure 1B for the stimulus protocol used. **(D)** Default model. **(E)** “Fixed pump model,” in which the Na^+^/K^+^-pump current *I*_pump_ is fixed to the initial steady-state value. **(F)** “Fully fixed K_M_ model,” in which the dynamics of the M-type K^+^ conductance was fixed to the initial steady-state value. **(G)** “Partially fixed K_M_ model,” in which the dynamics of the M-type K^+^ conductance was fixed only during +1 nA current injection. **(H–J)** Simulated changes of the spike count **(H)**, resting potential **(I)**, and input resistance **(J)** across trials, plotted with the corresponding interquartile ranges observed experimentally (blue shading). Colors in panels **(A–C)** and **(H–J)** indicate the different model conditions: (blue) default model; (green) “fixed pump model”; (black) fully fixed K_M_ model; and (red) “partly fixed K_M_ model.”

**FIGURE 4:**
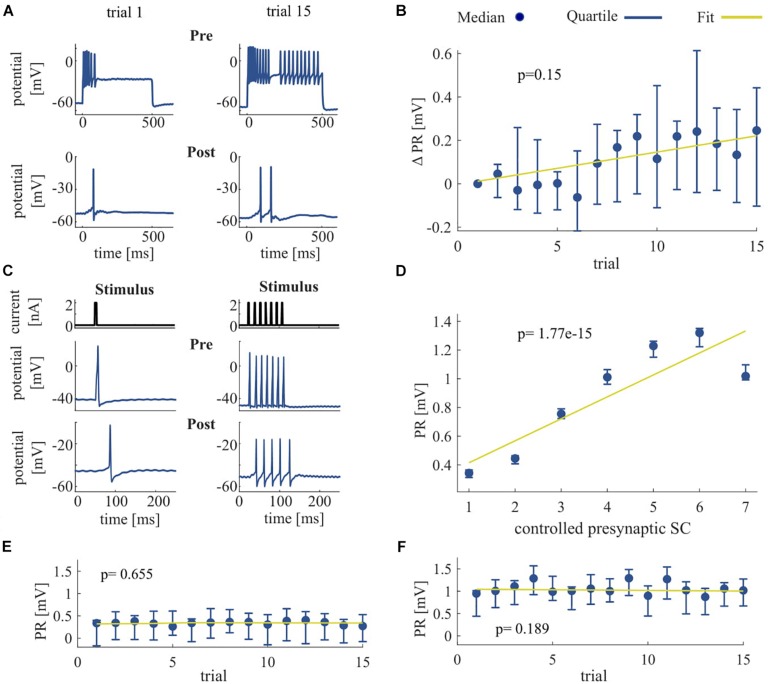
Effect of repeated somatic current stimulation on the communication between two ipsilateral T cells. **(A)** Representative responses of an intracellularly recorded T cell pair to a 500 ms long somatic current injection of 1 nA in two different trial repetitions. **(B)** PR difference from first trial [mV] as median and quartiles of all T cells (*N* = 11) vs. trials. **(C)** Representative responses of an intracellularly recorded T cell pair to one and seven short current pulses. **(D)** Dependency of PR as median and quartiles of all T cells (*N* = 14) on controlled presynaptic SC. **(E,F)** Dependency of PR as median and quartiles of all T cells (*N* = 14) for one **(E)** and for seven **(F)** presynaptic spikes vs. trials. The slope of the PR vs. trial was not significant for six out of seven conditions (1 pulse, *p* = 0.655; 2 pulses, *p* = 0.032; 3 pulses, *p* = 0.092; 4 pulses, *p* = 0.051; 5 pulses, *p* = 0.772; 6 pulses, *p* = 0.512; 7 pulses, *p* = 0.190). Linear fits were calculated for each cell individually and the obtained slopes were tested to differ significantly from 0 (*t*-test, α = 0.05). Yellow line indicates the average of the fits for all cells.

### Biological Neuron Model

We built a computational model of the leech T cell to investigate the physiological bases of the experimentally observed adaptive changes caused by repeated electrical stimulation. We used a single-compartment Hodgkin–Huxley-type neuron model modified with an additional Na^+^/K^+^-pump and a M-type slow potassium current (Table 1). We did not aim to create a more complex multi-compartment model, because we focus on the activity of T cells induced by somatic current injection and because the information on ion channel distribution over the T cell membrane is fundamentally lacking. This and other limitations of our modeling approach are addressed in section “Discussion.”

**TABLE 1:** T cell model equations.

**Variable**	**Equation**
Membrane potential *V*	$c_{m}\,\frac{d\,V}{d\,t}=I_{\text{Na}}+I_{K}+I_{M}+I_{L}+I_{\text{pump}}+I_{\text{inj}}$
Fast (transient) Na^+^ current *I*_Na_	*I*_Na_ = *g*_Na_⋅*m*^4^⋅*h*⋅(*E*_Na_−*V*)
Delayed rectifier K^+^ current *I*_*K*_	*I*_*K*_ = *g*_*K*_⋅*n*^2^⋅(*E*_*K*_−*V*)
M-type K^+^ current *I*_*M*_	*I*_*M*_ = *g*_*M*_⋅*z*^2^⋅(*E*_*K*_−*V*)
Leak current *I*_*L*_	*I*_*L*_ = *g*_*L*_⋅(*E*_*L*_−*V*)
Kinetic equations for channel variables (*x* = *m*, *h*, *n*, or *z*)	$\frac{d\,x\,\left(t\right)}{d\,t}=\frac{x_{\infty }\,\left(V\right)-x}{{\text{τ}}_{x}\,\left(V\right)}$
Steady state function for Na^+^ activation	$m_{\infty }\,\left(V\right)=\frac{1}{1+\exp\left(-\left(V+20\right)/8\right)}$
Time constant for Na^+^ activation (in ms)	$\tau_{m}\,\left(V\right)=0.75\cdot \left(\frac{2}{\exp\left(-\left(V+20\right)/16\right)+\exp\left(\left(V+20\right)/16\right)}+0.1\right)$
Steady state function for fast Na^+^ inactivation	$h_{\infty }\,\left(V\right)=\frac{1}{1+\exp\left(+\left(V+36\right)/5\right)}$
Time constant for fast Na^+^ inactivation (in ms)	$\tau_{h}\,\left(V\right)=7.5\cdot \left(\frac{2}{\exp\left(-\left(V+36\right)/10\right)+\exp\left(\left(V+36\right)/10\right)}+0.1\right)$
Steady state function for delayed rectifier K^+^ activation	$n_{\infty }\,\left(V\right)=\frac{1}{1+\exp\left(-\left(V+20\right)/8\right)}$
Time constant for delayed rectifier K^+^ activation (in ms)	$\tau_{n}\,\left(V\right)=4.0\cdot \left(\frac{2}{\exp\left(-\left(V+20\right)/16\right)+\exp\left(\left(V+20\right)/16\right)}+0.1\right)$
Steady state function for M-type K^+^ activation	$z_{\infty }\,\left(V\right)=\frac{1}{1+\exp\left(-\left(V+35\right)/3\right)}$
Time constant for M-type K^+^ activation (in ms)	$\tau_{z}\,\left(V\right)=450\cdot \left(\frac{2}{\exp\left(-\left(V+35\right)/6\right)+\exp\left(\left(V+35\right)/6\right)}+1.0\right)$
Na^+^/K^+^-pump current *I*_pump_	*I*_pump_ = −*I*_max_⋅*p*(*c*_Na_)
Na^+^/K^+^-pump activation function *p*(*c*_*Na*_)	$p\,\left(c_{\text{Na}}\right)={\left(\frac{1}{1+\exp\left(-\left(c_{\text{Na}}-18\right)/18\right)}\right)}^{3}$
Intracellular Na^+^ concentration *c*_*Na*_ (measured from rest)	$\frac{d\,c_{\text{Na}}}{d\,t}=\kappa_{\text{chan}}\cdot I_{\text{Na}}+3\,\kappa_{\text{pump}}\cdot I_{\text{pump}}$

*The factor 3 for the intracellular Na^+^ concentration change originates from the fact that three Na^+^ ions are exchanged with two K^+^ ions. The membrane potential *V* is in mV and the intracellular Na^+^ concentration *c*_*Na*_ is in mM. I_*inj*_ denotes injected current.*

#### Model Structure

Based on previous experimental recordings of leech neurons, we assumed a fast (transient) sodium channel with four activation and one inactivation gate, while the delayed rectifier potassium channel has two activation gates (Johansen, 1991). These channels are responsible for spike generation in response to positive current injections. The activity of the Na^+^/K^+^-pump was assumed to depend on the intracellular concentration of Na^+^ ions. Increase of intracellular Na^+^ due to repetitive spikes leads to the activation of the pump, which exchanges three intracellular Na^+^ ions with two extracellular K^+^ ions, resulting in the net negative current that hyperpolarizes the membrane potential (Forrest, 2014). The M-type K^+^ conductance, whose kinetics are much slower than the spike-generating conductances, cause the cessation of spiking during current injection in a voltage dependent manner (Yamada et al., 1998; Benda and Herz, 2003). In order to focus on the fundamental biophysical mechanisms of spike-rate adaptation caused by repetitive current injections, we used this minimalistic description of ion channels and pumps, although the leech T cell may express a number of other ionic conductances (see section “Discussion”).

#### Model Fitting

Since the operating voltage range of leech T cells is considerably more depolarized than those of neurons in most other animals, we needed to adjust the model functions (Table 1) and parameters (Table 2) to reproduce known T cell response characteristics (shown in sections “Results” and “Repeated Somatic Current Injection Induces Non-synaptic Plasticity”). Starting from previous measurements in leeches (reviewed, e.g., in Johansen, 1991), the ion channel kinetics were determined to fit our T cell data, including the RMP, spike threshold, single spike duration, and SCs. By using the standard membrane capacitance density of 1.0 μF/cm^2^, the effective size of the membrane and the leak conductance were determined. The membrane area we adopted is larger than what is expected solely from the size of the soma of a T cell, because the additional contribution of the dendritic processes near the cell body was considered.

**TABLE 2:** T cell model parameters.

**Parameter**	**Value**
Membrane surface area *S*_m_	15,000 μm^2^
Membrane capacitance density *C*_m_	1.0 μF/cm^2^
Membrane capacitance *c_m_* = *C*_m_*S*_m_	150 pF
Fast (transient) Na^+^ conductance density *G*_*Na*_	160 mS/cm^2^
Delayed rectifier K^+^ conductance density *G*_*K*_	8.0 mS/cm^2^
M-type K^+^ conductance density *G*_M_	4.0 mS/cm^2^
Leak conductance density *G*_*L*_	0.1 mS/cm^2^
Fast (transient) Na^+^ conductance *g*_*Na*_ = *G*_*Na*_*S*_m_	24 μS
Delayed rectifier K^+^ conductance *g*_*K*_ = *G*_*K*_*S*_m_	1.2 μS
M-type K^+^ conductance *g*_M_ = *G*_M_*S*_m_	0.6 μS
Leak conductance *g*_*L*_ = *G*_*L*_*S*_m_	0.015 μS
Na^+^ reversal potential *E*_*Na*_	+30 mV
K^+^reversal potential *E*_*K*_	−50 mV
Leak reversal potential *E*_*L*_	−15 mV
Maximum pump current *I*_*max*_	800 pA
Conversion factor from Na^+^ channel current into Na^+^ concentration κ_chan_	0.60 ⋅ 10^–6^ mM/pA ⋅ ms
Conversion factor from Na^+^/K^+^-pump current into Na^+^ concentration κ_pump_	0.12 ⋅ 10^–6^ mM/pA ⋅ ms

*Parameters were fitted to reproduce the experimentally measured T cell spike shape and to produce spike counts, resting potentials and input resistances in the interquartile range of the experimental observations of the 1st and 20th trial.*

There is no empirical data available for the ionic concentration in T cells at rest and at spiking. Therefore, we formulated the Na^+^/K^+^-pump model depending only on the change of Na^+^ concentration (denoted as *c*_Na_) from rest. In our simulations *c*_Na_ was assumed to be in a similar order as in Retzius cells (Deitmer and Schlue, 1983), namely a few tens of mM. Time-dependent changes of the intracellular Na^+^ concentration is caused by the Na^+^ currents through Na^+^ channels and Na^+^/K^+^-pumps. In order to separately simulate the contribution of Na^+^ channels and Na^+^/K^+^-pumps in *c*_Na_, we adopted two conversion factors (κ_chan_ and κ_pump_ in Table 2). Theoretically, this conversion factor is the reciprocal of the product between the Faraday constant and the effective volume relevant to the diffusion of Na^+^ ions (Barreto and Cressman, 2011). However, it is not simple to estimate these factors in a T cell, because of its complex neuronal morphology. If the diffusion of Na^+^ ions, which affects the activity of Na^+^/K^+^-pump, is not uniform but restricted to the vicinity of the membrane, the effective volume becomes smaller, leading to a larger value of the conversion factor. Therefore, we varied κ_chan_ and κ_pump_ in the range between 0 and 1.20 ⋅ 10^–6^ mM/pA ⋅ ms, and selected values that led to a stable decrease of membrane potential with repeated stimulations.

#### Comparison of Model Conditions

In order to investigate the effects of the Na^+^/K^+^-pump and putative M-type K^+^ (K_M_) conductance on the long-term change of repetitive spiking, we compared the following four conditions in the T cell model: (1) the “default model” introduced above with the functions and parameters fitted to our T cell data; (2) the “fixed pump” model in which the Na^+^/K^+^-pump current is fixed to the value at the initial steady-state obtained without any current injection; (3) the “fully fixed K_M_” model in which the M-type K conductance is fixed to the value at the initial steady-state obtained without any current injection; and (4) the “partially fixed K_M_” model in which the M-type K conductance is not allowed to change only during the +1 nA current stimulation in each trial. However, the K_M_ conductance may change normally as in the default model when there is no input, or during injected current of any other size than +1 nA (see Figure 1B for the stimulus protocol of each trial). The comparison of the default model and the partially fixed K_M_ model is expected to reveal how the dynamic property of the M-type K conductance may play a role during the specific current stimulation of +1 nA (500-ms long per trial).

## Results

### Repeated Somatic Current Injection Induces Non-synaptic Plasticity

High-frequency spike discharge leads to an intracellular accumulation of Na^+^ ions which activate Na^+^/K^+^-pumps and thereby provide a mechanism for intrinsic, activity-dependent regulation of excitability (Gulledge et al., 2013; Duménieu et al., 2015). We investigated the effect of high frequency spiking due to repeated stimulation with series of current pulse injections (500 ms duration, separated by 2500 ms break) into the T cell soma (Figure 1). Responses were analyzed for changes in the physiological properties SC, RMP, and cell IR. As can be seen in the representative intracellular response traces (Figures 2A,B), repeated current injection caused an increase in SC and in IR, while the RMP hyperpolarized.

These tendencies were seen in all recordings, even though the initial responses of individual T cells varied considerably in their SCs and the duration of their spiking activity (compare Figures 2A,B for examples of a slowly and a rapidly adapting initial response, see Figures 3H–J for the interquartile ranges of SC, RMP, and IR). The activity-dependent changes were found to be highly significant (*p* < 0.001) for all three physiological properties by testing if the slopes of linear regressions of individual cell responses differed from 0 (Figures 2C–E). This approach is not meant to imply that the relationships are linear, but only that SC and IR increased, and RMP hyperpolarized consistently in all 20 cells.

While Figure 2 shows the changes caused by repeated current injection, Figures 3H–J indicate the interquartile ranges of the observed measurements of SC, RMP, and IR. For an input current of 1 nA, the SC gradually increased from a median value of 14.5 in trial 1 to 36 in trial 20. Meanwhile the median RMP gradually hyperpolarized from −37.5 to −48.8 mV and the median T cell IR increased from 27.8 to 62.7 MΩ. The absolute changes of the physiological properties to a current injection of 1 nA, calculated as median and quartiles over cells, are shown in Table 3 (data for current injection of 0.5, 0.75, 1.25, and 1.5 nA are not shown, but followed the same trend).

**TABLE 3:** Absolute values for non-synaptic plasticity effects in T cells.

	**Trial 1**			**Trial 20**		
**Property**	**Q1**	**Median**	**Q3**	**Q1**	**Median**	**Q3**
SC [spikes/0.5 s]	7.5	14.5	24	27.5	36	42
RMP [mV]	–32.9	–37.5	–40.6	–44.5	–48.8	–52.9
Resistance [MΩ]	19.4	27.8	38.7	46.4	62.7	74.1

*Responses to 500 ms current injection of 1 nA were obtained in 20 cells.*

Summarizing, the RMP of T cells hyperpolarizes in response to repeated somatic current injection, while the SC increases. Usually, one would expect that the probability for action potential generation is decreased at hyperpolarized RMP (Hodgkin and Katz, 1949). However, the repetitive electrical stimulation also led to a substantial increase in cell IR, which cannot be explained by electrode clogging (see section “Experimental Design”). To investigate the reason of the experimentally observed increase in SC, we developed a Hodgkin–Huxley type neuron model, modified with an additional Na^+^/K^+^-pump and a M-type slow potassium current (Table 1).

### Sodium Pump and Slow Potassium Current Can Induce Non-synaptic Plasticity

A large number of previous studies already investigated how different forms of AHPs are affected by voltage- or calcium-dependent ionic conductances (reviewed in Vogalis et al., 2003; Larsson, 2013). The time scales of intracellular calcium buffering (Yamada et al., 1998; Goldman et al., 2001) and the activation/inactivation of calcium dependent potassium currents (Vogalis et al., 2003), however, is on the order of 0.1–1 s, which is several orders of magnitude faster than the activity-dependent long-term decrease of the membrane potential we focus on (which is on the order of a few tens of seconds; Figure 2A). In this slow potential change, the activity of the Na^+^/K^+^-pump is likely to be relevant (Barreto and Cressman, 2011; Forrest, 2014). Hence, we investigated the involvement of the Na^+^/K^+^-pump on the changes in the physiological properties of T cells, namely the hyperpolarization of the RMP accompanied by the increase of SC and IR. To address this question, we constructed a minimalistic Hodgkin–Huxley-type neuron model of a T cell incorporating the fast Na^+^, delayed rectifier K^+^, leak, and slow M-type K^+^ conductances as well as the Na^+^/K^+^-pump. Blue curves in Figure 3 show the responses of the default T cell model. To simulate the spiking responses of the T cell, we used the same protocol as for our experimental recordings with somatic current injections (Figure 1B). Parameters of the model were adjusted so that the simulated spike shape matched the time course of the original spike and the adaptive changes of the main features SC, RMP and IR stayed largely within the interquartile ranges of the original data (blue curves in Figures 3H–J). The increase of the pump current (Figure 3A, blue) was responsible for the decrease of the RMP from about −39 to −48 mV (Figure 3I, blue), because in each pump cycle three intracellular sodium ions were exchanged with two extracellular potassium ions, leading to a net negative current. The SC of the standard model increased over trials (Figure 3H), because of the voltage-dependent deactivation of the M-type K^+^ current (Figures 3B,C), which in early trials activates during the current injection and hinders the repetitive spiking. Additionally, the decrease of the M-type K^+^ conductance across trials also let to a decrease of the simulated IR from about 31–60 MΩ (Figure 3J), which resembled the empirical observations (Figures 2D,E), and also had a positive effect on SC.

In order to further investigate the roles of the Na^+^/K^+^-pump and the putative M-type K^+^ conductance, we fixed these current and examined the resulting changes of the model neuron responses from the default condition (see section “Comparison of Model Conditions” for more detail of the conditions compared). When the pump current was fixed to the initial steady-state value, the stimulus-induced hyperpolarization as well as the increase of SC and IR vanished (Figure 3E; compare the green and blue curves in Figures 3H–J). This suggests that the experimentally observed stable hyperpolarization of the RMP is mainly due to the increase of Na^+^/K^+^-pump current and that this hyperpolarization is the basis for all observed changes. When the M-type K^+^ conductance was fixed to the initial steady-state, while the pump was allowed to change, the IR did not change, indicating that the observed increase in IR can be attributed to the changes in the slow K^+^ current. In this model version, the SC decreased across trials (Figure 3F and black curves in Figures 3H,J), showing the intuitively expected effects of slow hyperpolarization (Figure 3I, black). This SC decrease was diminished when the M-type K^+^ conductance was kept unchanged only during the +1 nA stimulation (Figure 3G), while it could change in an activity-dependent way at all other times. In this model, the closing of the modeled M-type K^+^ channel still led to an increased IR (Figure 3J, red) and counteracted the reduced excitability due to hyperpolarization (compare black and red curves in Figure 3H). We also note, however, that the dynamic property of the M-type K^+^ current, which activates on the time scale of a few 100 ms, turned out to be critical for determining the duration of repetitive spiking (compare Figures 3D,G). Despite the realistic increase in IR, the number of spikes in the model version with partially fixed M-type K^+^ current were very large (Figure 3H, red), because spiking activity did not cease before the end of the stimulus (Figure 3G). These results suggest that the experimentally observed repetitive spiking behavior (and its cessation) of leech T cells is associated with a K_M_-like slow conductance, which affects the SCs more dynamically than the static increase of IR. In sum, our modeling results suggest that the inactivation of the slow K^+^ current plays a role in increasing the SC in an activity-dependent manner (Figure 3H). This inactivation is induced by the RMP hyperpolarization caused by the increased activity of Na^+^/K^+^-pumps (Figure 3I).

### Non-synaptic Plasticity Effects Postsynaptic Responses of Other T Cells

We investigated the signal transmission between two ipsilateral T cells. Our goal was to see how the activity changes that were induced by non-synaptic plasticity in an electrically stimulated T cell (presynaptic) affected the responses in a non-stimulated T cell (postsynaptic). We analyzed how the PR, consisting of postsynaptic potentials and potentially elicited spikes, changed over repeated stimulation of the presynaptic cell (Figure 4). As expected, the presynaptic SC increased with repeated current stimulation from a median of 6 spikes (Q1: 4.25/Q3: 20) in trial 1 to 20 spikes (Q1: 17.75/Q3: 21) in trial 15. Postsynaptically, we observed a tendency of increased PR size, but overall the slopes of the linear regressions of PR increases were not significantly different from 0 (*p* > 0.05, cf. Figure 4B), because the PR increased only in 9 out of 11 cells over trials. However, the median of the averaged PR increased from 0.77 mV (Q1: 0.30/Q3: 1.23) in trial 1 to 0.96 mV (Q1: 0.44/Q3: 1.67) in trial 15.

To clarify if the increase in PR observed in most of the T cell pairs was caused solely by the increase of presynaptic SC that reflects the non-synaptic plasticity in the presynaptic (stimulated) T cell, or rather the synaptic plasticity also played a role, we elicited in the presynaptic cell a fixed number of single action potentials (Figure 4C) and repeated the protocol over several trials. The PR size was found to depend highly significantly (*p* < 0.001) on the presynaptic SC (Figure 4D), because the linear regression yielded clearly positive slopes for all the cell pairs examined. The averaged PR increased from a median value of 0.34 mV in response to one spike (Q_1_: 0.31/Q_3_: 0.37) to 1.02 mV (Q_1_: 0.99/Q_3_: 1.10) in response to seven spikes. However, the PR size did not change significantly with stimulation repetitions in six out of seven conditions with fixed SC (Figures 4E,F), demonstrating that synaptic plasticity did not alter the PRs.

Summarizing, the increase in SC caused by non-synaptic plasticity in the repeatedly stimulated presynaptic cell in most T cell pairs was reflected by an increase in PR size in the postsynaptic cell. However, no indication for synaptic plasticity was found, because PR size stayed stable over trials, when the presynaptic SC did not change.

## Discussion

Many studies have reported the involvement of the Na^+^/K^+^-pump in activity-dependent synaptic plasticity in both vertebrates (Wyse et al., 2004) and invertebrates (Pinsker and Kandel, 1969; Scuri et al., 2007). Based on electrophysiological and modeling results, the present study showed that the Na^+^/K^+^-pump might also be involved in activity-dependent non-synaptic plasticity in leech sensory neurons. Repeated somatic T cell stimulation enhances Na^+^/K^+^-pump activity which gradually hyperpolarizes the RMP while the SC and IR increase (Figures 2, 3). Furthermore, we showed that this non-synaptic plasticity, rather than synaptic plasticity, leads to increased PRs in a second T cell on the same side of a segmental ganglion (Figure 4).

### Biophysical Mechanisms Underlying Activity-Dependent Non-synaptic Plasticity

Our experimental and modeling results indicate that T cells modify their responses depending on their previous activity. Repeated somatic T cell stimulation enhances Na^+^/K^+^-pump activity which gradually hyperpolarizes the RMP. This might result in the suppression of a slow K^+^ current, which leads to a higher IR and an increased SC. This putative K_M_-current is involved in producing burst-patterns in many neuronal systems by raising the threshold for firing an action potential (Benda and Herz, 2003). Some of the K_M_-channels are open at rest and they are even more likely to be open during depolarization, causing spike responses to stop before the end of the stimulation. But when the cell hyperpolarizes, this slow K^+^ channel closes and the neuron fires in a more tonic manner. Especially in early trials, many of the recorded T cells showed cessation of spikes well before the end of the current pulse period. While the experimentally observed increase in IR could be one of the causes for the increase in SC, it did not lead to the cessation before the end of the stimulus. The changes in repetitive spiking behavior could be explained by the dynamical changes of the putative K_M_ current. Further experimental and modeling studies, e.g., on the effects of varied intervals between stimulus applications, are needed to understand the biophysical mechanisms of non-synaptic plasticity in more detail.

Leech sensory cells express several different ion channels (Johansen, 1991; Kleinhaus and Angstadt, 1995; Gerard et al., 2012), most of which we did not include in our model for the sake of simplicity. Sodium-dependent K^+^ channels as they were found in leech pressure cells (Klees et al., 2005), for example, might also affect T cell spiking in an activity dependent manner. Additionally, we assumed that the kinetics of the slow K^+^ channels were only voltage dependent. However, the activity of M-type K^+^ channels was reported to be affected also by the intracellular concentration of ATP (Simmons and Schneider, 1998). Since ATP is consumed through Na^+^/K^+^-pump cycles, this additional mechanism could further modulate the spike rate adaptation.

One important discrepancy between our model and the experimentally measured neuronal response was the difference in spike amplitudes. This difference might imply that the spike initiation site of the real T cell, responding to somatic stimulation, is not located exactly in the cell body. The recorded somatic spikes may only reflect the propagated action potentials that are generated elsewhere. Non-uniform distribution of ion channels in the T cell was found in previous studies: for example, T cells do have calcium-dependent K^+^ channels (Kleinhaus and Angstadt, 1995) while their cell bodies largely lack voltage-dependent calcium channels (Valkanov and Boev, 1988; Johansen, 1991). To replicate the observed membrane potential changes, we had to adopt different factors (κ_chan_ and κ_pump_ in Table 2) that convert Na^+^ channel currents and Na^+^/K^+^-pump currents into the intracellular Na^+^ concentration; the difference between the two conversion factors possibly implies that these current sources may exist in different locations. In addition, recorded spike heights did not decrease over trials (Figure 2A), making contrast to the naive expectation that the increased intracellular Na^+^ concentration should lead to the decrease of the sodium reversal potential. Overall, these observations support the hypothesis that somatic spikes of the T cell may actually be generated not directly within the cell body but somewhere nearby in the cell processes.

In order to experimentally confirm the role of Na^+^/K^+^-pumps and M-type K^+^ channels in the change of IR and the spike rate adaptation of T cells, their pharmacological blockade would be necessary. Such an experiment might not be straightforward, because, in leech neurons, conventional blockers do not always suppress ionic currents as expected (Johansen and Kleinhaus, 1986). Furthermore, leech neurons are packed in segmental ganglia, where the extracellular space is limited. To better simulate the effect of limited resources, a model would need to consider both intracellular and extracellular ionic concentrations (Hübel and Dahlem, 2014). To theoretically investigate the possible roles of spatially distributed ion channels and pumps in neuronal information processing, multicompartment models would be required (Cataldo et al., 2005; Kretzberg et al., 2007). In the present study, however, we did not aim to create a multicompartment model for several reasons. First, the main focus of our modeling was to investigate the activity-dependent long-term activity change of T cells induced by repetitive somatic current injection. Second there is almost no information available on the spatial distribution of ion channel over the T cell membrane that can be used for creating a multicompartment model. Third, we have no data recorded from the dendritic processes of T cells, which are essential for testing the simulated initiation and conduction of peripherally induced spikes.

### T Cell Function and Interaction in the Network

Leeches respond to identical stimulation with multiple distinct reactions, which inspired the discussion of behavioral choice in the leech (Kristan et al., 2005; Baljon and Wagenaar, 2015). These behavioral alternatives are triggered by different patterns in neuronal activity. Due to the small number of neurons, multifunctional cells play a particular role in shaping these patterns (Kristan et al., 2005; Briggman and Kristan, 2006; Frady et al., 2016). We assume that T cells are such multifunctional players in a network that controls the response to a light touch on the leech’s skin. The activated segment produces shortening on the stimulated and lengthening on the opposite side of the body wall (Kristan, 1982; Kristan et al., 1982). This local bend response is one of the fastest behaviors of the leech, with muscle movements starting only 200 ms after stimulus onset (Kristan et al., 2005). T cells respond very fast to tactile stimulation and give input to several local bend interneurons before P cell neuron spikes arrive at the central ganglion (Kretzberg et al., 2016). However, T cells are not capable of eliciting a behavior on their own (Kristan, 1982; Fathiazar et al., 2018). We assume that they activate with their very fast responses a modulatory network that regulates the gain of behaviors. Frady et al. (2016) have identified such a “preparatory network” of neurons that starts to react with a very short latency prior to the production of multiple behaviors, including local bending and whole-body shortening. After repeated stimulation causing non-synaptic plasticity, the enhanced activity might enable the T cells to transfer the animal into a state that prepares the muscles for a rapid start of a behavioral response by shifting the threshold for firing action potentials.

T cells form synaptic connections with each other that have both an electrical and a chemical component (Nicholls and Baylor, 1968b; Li and Burrell, 2008). Electrical synapses have shorter latencies compared to chemical synapses (Nicholls and Purves, 1970), and such fast-acting synaptic inputs could facilitate rapid transmission of sensory information to the preparatory network. Our experiments revealed that the increase in SC by non-synaptic plasticity also increases the PR in other T cells, which may include postsynaptic spiking. These postsynaptic changes appear to be a mere reflection of the activity-dependent changes in the presynaptic T cell. This finding might be an alternative interpretation to the conclusion of previous studies that the Na^+^/K^+^-pump is involved in activity-dependent synaptic plasticity between two ipsilateral T neurons (Catarsi and Brunelli, 1991; Catarsi et al., 1993; Scuri et al., 2002, 2007; Lombardo et al., 2004). Additionally, T cells have a strong synaptic connection to the S cell, a prominent element of the preparatory network (Burrell and Sahley, 2004). Each ganglion contains a single unpaired S cell, which forms strong electrical connections to the S cells in adjacent ganglia (Sahley et al., 1994). This network is reminiscent of a fast-conducting system (Mistick, 1978), but its causal role in any behavior is unclear (Sahley et al., 1994). To determine more neurons and their interactions involved in this preparatory network, VSD imaging with a double-sided-microscope would be an appropriate method (Tomina and Wagenaar, 2018). It would allow us to directly analyze functional relationships between neurons located on opposite surfaces and detect both action potentials and sub-threshold excitatory and inhibitory synaptic potentials (Miller et al., 2012).

In many vertebrate neurons, spikes are generated in the axon hillock, the part of the cell body that connects to the axon (Clarac and Cattaert, 1999). However, many invertebrate neurons are unipolar, meaning that dendrites and the axon are not clearly separated, but form a continuum of processes (Rolls and Jegla, 2015). Leech T cells send several long-range touch-sensitive dendritic processes to their receptive fields in the skin several millimeters away (Nicholls and Baylor, 1968b). Additionally, they arborize centrally to also reach the skin via both adjacent ganglia, leading to an even more extended receptive field (Yau, 1976). When the skin is touched lightly, T cells respond to the rate of skin indentation generating transient, rapidly adapting responses at stimulus onset and offset (Carlton and McVean, 1995; Kretzberg et al., 2016; Pirschel and Kretzberg, 2016; Pirschel et al., 2018). The action potentials are generated in the periphery and propagate through the ipsilateral nerve roots toward the soma. Moreover, spikes induced by central synaptic input can also travel in the opposite direction toward the periphery (Yau, 1976). Burgin and Szczupak (2003) and Kretzberg et al. (2007) suggested that leech T neurons may have at least two spike initiation zones with the different computational tasks of encoding touch stimuli in the periphery and processing synaptic inputs in the central ganglion. A similar anatomy was found in the LG motor neuron of the stomatogastric nervous system of the crab. It has a large soma with a nearby spike initiation zone and projects via dendritic processes to the periphery, where it also can initiate spikes up to 2 cm away from the soma (Meyrand et al., 1992).

In addition to the mutual T cell connections, the central part of the T cells receives polysynaptic inputs from the other mechanoreceptors, the P and N cells. The postsynaptic potentials induced by these mechanoreceptor inputs can be excitatory, inhibitory, or a combination of both, indicating two interneuron pathways between the other mechanoreceptors and the T cells (Burgin and Szczupak, 2003). Hence, Na^+^/K^+^-pump activation in the context of non-synaptic plasticity probably shifts the membrane potential relative to the reversal potentials of these synapses, which could lead to an increase of the excitatory and a decrease of the inhibitory components of these postsynaptic potentials. In consequence, two complementary mechanisms following repeated T cell stimulation could increase the probability for centrally elicited spike reactions to synaptic inputs from the other mechanoreceptors: The hyperpolarization could increase excitatory postsynaptic potential size and at the same time deactivate a slow K^+^-current, leading to an increase in SC. Therefore, the activity-dependent non-synaptic plasticity we described in this study could act like a gain mechanism. It could serve the purpose of tuning the T cells – and maybe in consequence also postsynaptic cells in the preparatory network – in an activity-dependent way to the combination of two computational tasks. Depending on the previous activity, T cells could react more or less to the inputs from the other mechanorecepors, and combine these synaptic responses with the spikes that encode light skin stimulation, which are elicited by T cell processes in the periphery.

### Outlook

The local bend, one of the fastest movements in the leech, is initiated by sensory stimulation of the body wall. The morphology and response properties of the mechanosensory touch (T) cells suggest that T cells have at least two spike initiation zones, one in the periphery and one in the central ganglion. While spikes generated in the periphery should faithfully represent mechanical skin stimulation, the central part of the T cell should integrate and react to synaptic inputs from all three mechanoreceptor types. The activity-dependent non-synaptic plasticity introduced in this study could serve as a mechanism to tune the interaction of both spike initiation zones and their computational tasks. To test this hypothesis, further experiments are necessary to study to which extend repeated skin stimulation also triggers non-synaptic plasticity and how both spike initiation zones interact in these situations. If our hypothesis is correct, this multi-tasking ability and its activity-dependent tuning could make the T cell a key player in a fast-conducting preparatory network that regulates the gain of behaviors. The leech nervous system allows to study such network effects by further voltage-sensitive dye imaging of the ganglion during repeated skin stimulation and/or current injection to the T cell soma (Kretzberg et al., 2016; Fathiazar et al., 2018). Hence, despite the small number of neurons, the leech tactile system might be suitable for studies on general mechanisms underlying the flexibility of neural activity and behavior. As mechanoreceptors of leeches and humans share several fundamental properties (Baca et al., 2005; Pirschel and Kretzberg, 2016), our results might inspire studies in the field of prosthetics and artificial skins (Kim et al., 2014).

## Data Availability Statement

The datasets generated for this study are available on request to the corresponding author.

## Author Contributions

JK and SM planned the study and designed the figures. SM performed the experiments, analyzed the data, and drafted the text. GA did the neuron modeling. All authors contributed to writing the manuscript and interpreting the results.

## Conflict of Interest

The authors declare that the research was conducted in the absence of any commercial or financial relationships that could be construed as a potential conflict of interest.
